# Modeling Cell Energy Metabolism as Weighted Networks of Non-autonomous Oscillators

**DOI:** 10.3389/fphys.2020.613183

**Published:** 2021-01-28

**Authors:** Joe Rowland Adams, Aneta Stefanovska

**Affiliations:** Physics Department, Lancaster University, Lancaster, United Kingdom

**Keywords:** networks, oscillations, metabolism, cells, non-autonomous oscillators, Kuramoto oscillators, non-linear dynamics, synchronization

## Abstract

Networks of oscillating processes are a common occurrence in living systems. This is as true as anywhere in the energy metabolism of individual cells. Exchanges of molecules and common regulation operate throughout the metabolic processes of glycolysis and oxidative phosphorylation, making the consideration of each of these as a network a natural step. Oscillations are similarly ubiquitous within these processes, and the frequencies of these oscillations are never truly constant. These features make this system an ideal example with which to discuss an alternative approach to modeling living systems, which focuses on their thermodynamically open, oscillating, non-linear and non-autonomous nature. We implement this approach in developing a model of non-autonomous Kuramoto oscillators in two all-to-all weighted networks coupled to one another, and themselves driven by non-autonomous oscillators. Each component represents a metabolic process, the networks acting as the glycolytic and oxidative phosphorylative processes, and the drivers as glucose and oxygen supply. We analyse the effect of these features on the synchronization dynamics within the model, and present a comparison between this model, experimental data on the glycolysis of HeLa cells, and a comparatively mainstream model of this experiment. In the former, we find that the introduction of oscillator networks significantly increases the proportion of the model's parameter space that features some form of synchronization, indicating a greater ability of the processes to resist external perturbations, a crucial behavior in biological settings. For the latter, we analyse the oscillations of the experiment, finding a characteristic frequency of 0.01–0.02 Hz. We further demonstrate that an output of the model comparable to the measurements of the experiment oscillates in a manner similar to the measured data, achieving this with fewer parameters and greater flexibility than the comparable model.

## 1. Introduction

Analyzing the energy metabolism of a cell can be key to understanding more about its functions, states and health. A malfunctioning metabolism is indicative of a wide range of pathological states, from diabetes, to Alzheimer's, to cancer (Seyfried and Shelton, 2010; Akter et al., 2011; Bosco et al., 2011; Kembro et al., 2018). A healthy metabolism also plays a significant role in other higher order processes through its production of adenosine triphosphate (ATP), which, for example, allows the generation of a membrane potential. The membrane potential is itself crucial for a variety of functions, including maintaining the cell's structural integrity and the firing mechanism of neurons (Macknight, 1988; Kuwahata, 2004).

Cellular ATP is generated mainly through glycolysis in the cytosol, consuming glucose, and oxidative phosphorylation (OXPHOS) in the mitochondria, consuming oxygen (Wilson, 2017; Chaudhry and Varacallo, 2020). Like many biological processes, experimental observations have established that these reactions are oscillatory (Betz and Chance, 1965; Jung et al., 2000; Kennedy et al., 2002; Richard, 2003; Tu et al., 2005; Jafri, 2006; Olsen et al., 2009; Ganitkevich et al., 2010; Kurz et al., 2010a; Ozalp et al., 2010; Bechtel and Abrahamsen, 2011; Porat-Shliom et al., 2014; Thoke et al., 2015; Lancaster et al., 2016). Not only this, but there is further evidence to suggest these oscillations may be non-autonomous (O'Rourke et al., 1994; Tu and McKnight, 2006; Kurz et al., 2010b; Battle et al., 2016; Rupprecht and Prost, 2016; Amemiya et al., 2017): that their frequencies vary over time. Modeling this behavior is a challenge for many traditional techniques, which often rely on perturbations of a steady state to give rise to oscillations, and the addition of noise to simulate non-autonomous variation. We present here an alternative approach to modeling non-autonomous oscillations in living systems, and what we can learn from such models.

The time variation of biological oscillations is often neglected, even where the existence of oscillations is acknowledged. Many modeling theories assume this variation to be due to noise, arising either from experimental methods or from the complexity of the system's interactions, and therefore that it can be averaged out when considered over asymptotic time. Time sensitive analysis of such data can show that the variation in a process's oscillations, induced by interactions with its surroundings and otherwise, is often deterministic (Lucas et al., 2018, 2019). Lucas et al. (2018) and Lucas et al. (2019) further showed that allowing for this deterministic variation in a model's architecture, and analyzing it over the finite time scales within which biological systems actually exist, can reveal dynamics that would be missed in a solely asymptotic approach. In particular, an intermittent synchronization, where oscillators are synchronized at some times and not others, without any change of parameters, can only exist when oscillations are allowed to be non-autonomous and only found when they are analyzed with finite time techniques.

The origins of our cellular metabolism model lie in the work of Lancaster et al. where glycolysis and OXPHOS are each represented by bi-directionally coupled non-autonomous Kuramoto oscillators (Kuramoto, 1984), and each driven by a non-autonomous oscillator depicting the supply of glucose and oxygen, respectively (Lancaster et al., 2016). This model was built on the theory of chronotaxicity (Suprunenko et al., 2013), which studies the effects of non-autonomicity to stabilize oscillators in spite of perturbations, an important ability for biological processes.

However, like most biological processes, neither glycolysis nor OXPHOS are a single process, but many (Kurz et al., 2017, 2018; Cortassa et al., 2018; Kembro et al., 2018; Vetter et al., 2020). Glycolysis occurs distributed throughout the cytosol, while OXPHOS is localized within the many mitochondria of the cell. These processes further communicate between themselves as well as one another. Glycolysis was found to signal inter- and intra-cellularly through the exchange of acetaldehyde (Richard, 2003; Madsen et al., 2005; Weber et al., 2012), while OXPHOS is thought to interact in many possible ways, including molecular exchange, common regulation and inter-mitochondrial nano tunnels (Kohnhorst et al., 2017). Here, we extend the Lancaster et al. (2016) model to consider glycolysis and OXPHOS as all-to-all coupled networks of oscillators. These networks are furthermore weighted such that oscillators closer to each other around a ring are connected more strongly than those further from one another, to reflect the nature of molecular exchange over a range of distances. We also draw from the work of Petkoski and Stefanovska (2012); Petkoski et al. (2013). who introduced a method of phase coupling through mean fields of ensembles of oscillators.

We present here a summary of the Lancaster et al. (2016) non-autonomous oscillator model for cell energy metabolism the details of its adaptation to weighted networks of oscillators, informed throughout by our alternative approach to modeling oscillating living systems. We will discuss further the analysis that had and can be done on these models, and what they can reveal about the biology of the cellular production of ATP and its role in wider processes.

## 2. Materials and Methods

Our modeling approach consists of four main principles, which are summarized in Table 1. We consider the cell to be the minimal functioning biological unit: processes within the cell cannot be isolated and still function and more macroscopic functions can be built from a cellular level, but the cell itself can survive provided the appropriate molecular supply in its environment. It is crucial however that the cell is able to expel waste and absorb needed molecules. This makes the cell a thermodynamically open system: matter and energy must cross its boundaries in order for the cell to survive. One of the principles of our approach is therefore to treat the cell and its internal processes as open, constructing a model that does not impose a constant mass on the system. While many models make mass their subject, it is much easier to achieve the aim of an open system by focusing the phase of the processes instead, and so in our model we consider the phase of oscillations.

**Table 1:** Summary of the principles informing our modeling approach contrasted to those of mainstream approaches.

**Mainstream principles**	**Our principles**
Open systems can be modeled as perturbed closed systems	Open systems can only be fully represented by open models
Oscillations result from instability of a dynamical system	Oscillations are inherent to the dynamics of open systems. Living systems continuously exchange energy and matter with the environment and each process is characterized by self-sustained oscillations on a certain time-scale
Non-linear systems can be recombined from linear systems	Non-linear systems are best understood by non-linear models
Time variation in living systems is often due to noise, and can be averaged out over asymptotic time	Time variation in living systems is often deterministic, and must be modeled as non-autonomous to reflect the full system dynamics

Our second principle is to treat oscillating systems as not just a temporary perturbation from a steady state, but as fundamentally defined by their oscillations. We therefore do not construct our model as a non-oscillating set of processes and subsequently find sets of parameters that induce oscillations, but set oscillations as the foundation of the model by representing each process with a phase oscillator. Cellular processes are also inevitably characterized by their non-linearity (Carballido-Landeira and Escribano, 2016), and modeling these non-linearities is essential to understanding their dynamics. We therefore use Kuramoto oscillations to model these interactions.

Unlike theories that assume variations in the features of these oscillations, in particular frequency, are due solely to noise endemic to the complexity of biological systems, we treat much of these observable variations as deterministic. Our modeling approach to these systems is to represent them as non-autonomous Kuramoto phase oscillators.

### 2.1. Cell Energy Metabolism

**Figure 1:**
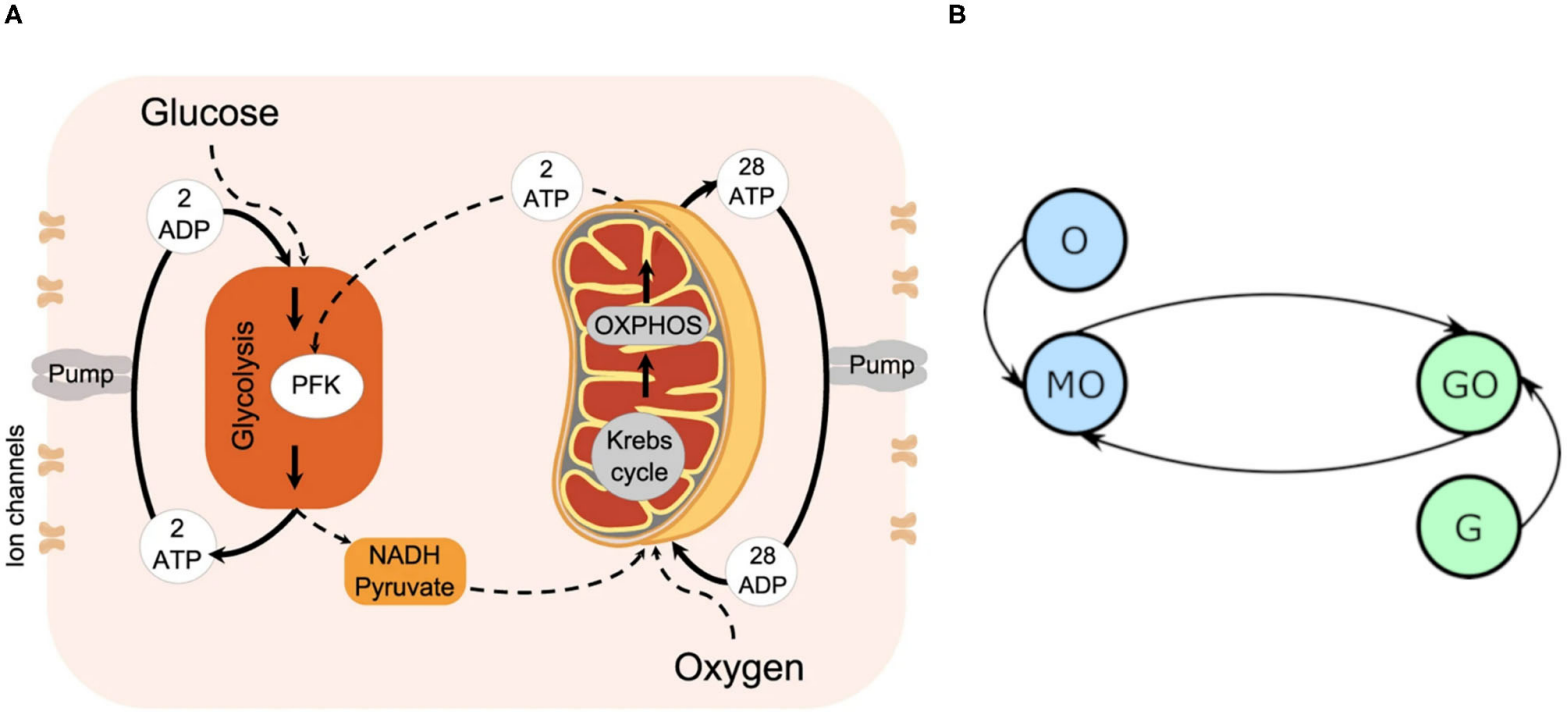
**(A)** The cellular energy metabolism considered in the model, reprinted with permission from Lancaster et al. (2016). **(B)** An oscillator model diagram of **(A)**, where each circle represents an oscillator, and each line a coupling. MO denotes the mitochondrial oscillator, GO the glycolysis, G the glucose driving, and O the oxygen.

The biological system as considered in this model is summarized in Figure 1A, and represented in the model's format in Figure 1B. It is constituted by four key processes: glycolysis, converting glucose, ATP and ADP into NADH, pyruvate and ATP, OXPHOS, converting oxygen, NADH and pyruvate into ATP, and the supplies of glucose and oxygen. The main purpose of this mechanism is the creation of ATP, which is primarily used to fuel ion pumps. Ion pumps actively transport ions across the cell's boundary against the electrochemical gradient, without which the cell would be forced to maintain an ionic equilibrium with its surroundings. Instead, the cell is able to accept the ions it needs for survival, and prevent itself from being flooded with an unhealthy quantity. Neuronal firing also relies on the ability of ion pumps to dramatically and rapidly change the balance of ions between the cell interior and exterior: the process is triggered only once the cell's membrane potential crosses the action potential threshold, typically requiring a change of some 100mV (Catterall et al., 2012).

Communication between the metabolic processes is also well-established (Richard, 2003; Madsen et al., 2005; Weber et al., 2012; Kohnhorst et al., 2017). Glycolysis enzymes exist all around the cytosol, each facilitating an element of the wider glycolytic reaction. Not only do these distributed enzymes rely on regulation and supply common to them all, but the exchange of acetaldehyde molecules has been observed to drive coherence between glycolytic processes. Mitochondria, housing the OXPHOS process, exist in a more fixed state than the glycolysis enzymes of the cytosol, but are similarly thought to mutually organize their processes for the efficient running of the cell. Mapping precisely the exact positions and connections of these processes however would be challenging, if not impossible. In our model we therefore focus on the importance of molecular exchanges in their communication, and the diffusive nature of these exchanges making distance a key consideration. Hence, we have assumed all-to-all coupled networks, but weighted these connections such that if were they considered around a ring, coupling strength would decrease the further apart any two given oscillators were.

### 2.2. Defining the Model

**Figure 2:**
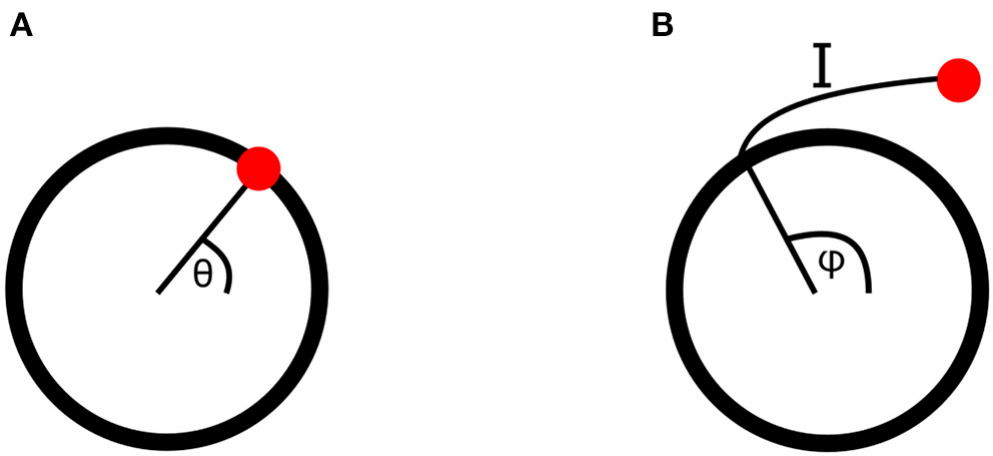
**(A)** An oscillatory cycle in phase space, at a phase value of θ. **(B)** A point perturbed from an oscillatory cycle, returning along isochron I to the cycle at a point with phase φ. The perturbed point is therefore also assigned the phase φ.

Each of these four metabolic processes is represented by a Kuramoto oscillator. Kuramoto oscillators are a type of non-linearly interacting phase oscillators, which are a reduction of ordinary differential equations featuring self-sustaining oscillations from many degrees of freedom to just one: the phase of the oscillation. The phase of an oscillator is defined as its position along its cycle at a given time. This cycle can be represented in phase space, as shown in Figure 2A, where the meaning of any particular phase value can easily be seen.

Here phase has only been defined on the cycle of the oscillator equation. However, when oscillators interact or are driven by external forces, they will be perturbed away from this cycle. The phase in the vicinity of the cycle must therefore also be defined, which can be done for stable oscillators using isochrons. When a stable oscillator is perturbed its phase will initially leave its cycle, but will return to it over time if not further perturbed. Isochrons connect the point to which a phase is perturbed to the point on the cycle it will first return to after the decay of the perturbation, assigning both the same phase value. This is demonstrated in Figure 2B. In order to remain in this region of attraction of the cycle, where isochrons can be used, the perturbations must be sufficiently weak, placing constraints on the strength of couplings between oscillators and drivers (Pikovsky et al., 2001; Strogatz, 2001).

This definition requires further extension to allow for the phases of non-autonomous oscillators. As the frequency, also known as the velocity of the phase, of the oscillator changes at each moment in time the system is transformed from one autonomous system to another. To maintain a consistent definition of phase across these systems, we must require that each system resides in the region of attraction of the one proceeding it, in order to use the same reasoning as the isochrons of perturbations. As with the weak coupling requirements of interactions, this definition constrains the system to only small changes in the frequency of oscillation from second to second (Kloeden and Rasmussen, 2011).

This theory was applied to the biology of cellular ATP production by Lancaster et al. (2016) in the following equations for the oscillators' phases

(1) $$\begin{array}{l} {\overset{∙}{\theta }}_{GO}=\omega_{GO}+\epsilon_{1}\sin\left(\theta_{GO}-\theta_{MO}\right)-\epsilon_{4}\sin\left(\theta_{GO}-\omega_{G}t\right)+\sigma \eta \left(t\right)\\ {\overset{∙}{\theta }}_{MO}=\omega_{MO}-\epsilon_{2}\sin\left(\theta_{MO}-\theta_{GO}\right)-\epsilon_{3}\sin\left(\theta_{MO}-\omega_{O}t\right)+\sigma \eta \left(t\right), \end{array}$$

where the subscript *GO* represents the glycolytic oscillator, *MO* the OXPHOS, *G* the glucose driving and *O* the oxygen. ω_*X*_ is the frequency of oscillator *X*, ϵ the relevant oscillator coupling strength, θ_*X*_ the phase, *t* time, η(*t*) a noise term and σ the scaling parameter of the noise. These are hence two oscillators as described above, coupled to one another and their respective metabolic drivers, with their frequency rendered non-autonomous by the addition of a time-dependent noise parameter.

**Figure 3:**
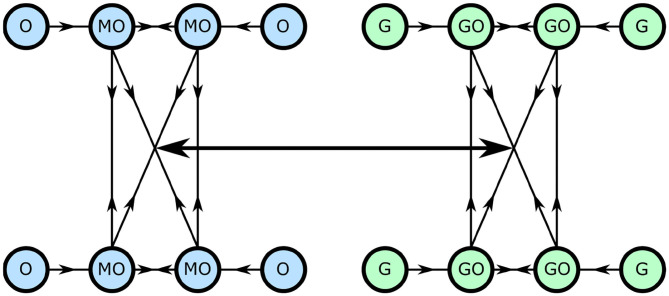
The network cellular metabolism model, with each circle representing an oscillator and each line a coupling.

We convert this model to now consist of networks of oscillators, weighted such that neighbors around a ring interact with a maximal coupling strength, and those opposite with a minimal strength. This is shown diagrammatically in Figure 3.

We also consider, instead of the stochastic non-autonomicity in Lancaster et al. (2016), a deterministic variation of the oscillation frequencies. This gives the glycolysis and OXPHOS phase equations as

(2) $$\begin{array}{l} {\overset{∙}{\theta }}_{GOni}=\frac{K_{GO}}{N}\overset{N}{\underset{j=1}{\sum }}W_{ij}\sin\left(\theta_{GOj}-\theta_{GOi}\right)\\ {\overset{∙}{\theta }}_{MOni}=\frac{K_{MO}}{M}\overset{M}{\underset{j=1}{\sum }}W_{ij}\sin\left(\theta_{MOj}-\theta_{MOi}\right), \end{array}$$

respectively, where θ_*GOni*_ is the phase of the oscillator *i* due to network interactions, *N* is the number of glycolytic oscillators, *M* the number of OXPHOS oscillators, *K*_*X*_ the relevant network coupling strength and *W*_*ij*_ the weighting function between oscillators *i* and *j*.

The oscillators are organized into all-to-all couple networks, with a certain weight applied to each coupling. Each oscillator is further assigned an index, to create a ring structure where oscillator *i* and *i*+1 are considered neighbors, as are the first oscillator, index 1, and the final, index *N*. The weight of the coupling between these oscillators is determined by their indices, such that the larger the difference between the indices, the smaller the weighting of their coupling. This weighting function is defined, for $i\leq \frac{N}{2}$, as

(3) $$\begin{array}{l} W_{ij}=\{\begin{array}{l} \frac{W}{\left|i-j\right|},\text{ for}\;j\in \left[1,i+\frac{N}{2}-1\right]\\ \frac{W}{\left|j-N-i\right|},\text{for}\;j\in \left[i+\frac{N}{2},N\right], \end{array} \end{array}$$

and for $N\geq i>\frac{N}{2}$ as

(4) $$\begin{array}{l} W_{ij}=\{\begin{array}{l} \frac{W}{\left|i-j\right|},\text{ for}\;j\in \left[i-\frac{N}{2}+1,N\right]\\ \frac{W}{\left|j+N-i\right|},\text{for}\;j\in \left[1,i-\frac{N}{2}\right], \end{array} \end{array}$$

where *W* is a constant to be chosen.

The glucose and oxygen drivers are

(5) $$\begin{array}{l} {\overset{∙}{\theta }}_{GOGi}=\epsilon_{G}\sin\left(\theta_{GOi}-\theta_{Gi}\right)\\ {\overset{∙}{\theta }}_{MOOi}=\epsilon_{O}\sin\left(\theta_{MOi}-\theta_{Oi}\right), \end{array}$$

where θ_*GOGi*_ is the phase of glycolysis oscillator *i* due to glucose coupling. The inter-network interactions arise through coupling each network to the mean field of the other (Strogatz and Mirollo, 1991; Petkoski and Stefanovska, 2012; Petkoski et al., 2013). This mean field arises as the average of each individual oscillation, characterizing their collective state. It can be defined through the Kuramoto order parameter, $r_{X}e^{i\text{Ψ}}=\frac{1}{N}\overset{N}{\underset{k=1}{\sum }}e^{i\theta_{Xk}}$, where Ψ is the phase of the mean field. *r*_*X*_ = 1 hence indicates a totally ordered network with all oscillators at the same phase of their cycle, while *r*_*X*_ = 0 represents a totally disordered network. The inter-network equations therefore are

(6) $$\begin{array}{l} {\overset{∙}{\theta }}_{GOMOi}=F_{GO}r_{MO}\sin\left({\text{Ψ}}_{MO}-\theta_{GOi}\right)\\ {\overset{∙}{\theta }}_{MOGOi}=F_{MO}r_{GO}\sin\left({\text{Ψ}}_{GO}-\theta_{MOi}\right), \end{array}$$

where *F*_*X*_ is the inter-network coupling strength and the average phase of network *X* is ${\text{Ψ}}_{X}=\frac{1}{N}\overset{N}{\underset{i=1}{\sum }}\theta_{Xi}$.

The four governing differential phase equations are hence

(7) $$\begin{array}{l} \;{\overset{∙}{\theta }}_{Gi}=\omega_{Gi}\left(t\right)\\ \;{\overset{∙}{\theta }}_{Oi}=\omega_{Oi}\left(t\right)\\ {\overset{∙}{\theta }}_{GOi}=\omega_{GOi}\left(t\right)+{\overset{∙}{\theta }}_{GOni}-{\overset{∙}{\theta }}_{GOGi}+{\overset{∙}{\theta }}_{GOMOi}\\ {\overset{∙}{\theta }}_{MOi}=\omega_{MOi}\left(t\right)+{\overset{∙}{\theta }}_{MOni}-{\overset{∙}{\theta }}_{MOOi}-{\overset{∙}{\theta }}_{MOGOi}, \end{array}$$

where ω_*Gi*_(*t*) = ω_*G*_ + *A*_*G*_sin(ω_*Gm*_*t* + *t*_*i*_), and ω_*O*_(*t*), ω_*GO*_(*t*) and ω_*MO*_(*t*) have equivalent expressions for their respective elements, is the time-varying natural frequency of each oscillator *i*. In this paper we use the deterministic variation formulation for these frequencies, but any other time varying formulation, such as random noise, are also valid methods provided that the variation is slow. ω_*G*_ is the mean frequency around which the non-autonomous frequency is modulated, *A*_*G*_ is the amplitude of modulation of the frequency, ω_*Gm*_ is the frequency of modulation and *t*_*i*_ is a perturbation of the modulation in time, taking a random number between 0 and $\frac{1}{\omega_{Gm}}$s. This perturbation ensures a distribution of frequencies within each element, while assigning the oscillators the same mean frequency and deterministic cycle of modulation.

### 2.3. Analyzing Synchronization

The phenomenon of synchronization between oscillators is a key part of understanding their dynamics. Oscillators can be considered synchronized when the difference between their phases remains constant. This is well-established in the context of permanent synchronization, where the phase difference between two oscillators does not ever change unless the parameters of the system change or a new influence is introduced (Pikovsky et al., 2001; Strogatz, 2001). Lucas et al. (2018) and Lucas et al. (2019) however found a different form of synchronization, intermittent, where a pair of oscillators can transition repeatedly between synchronized and unsynchronized states without the system being changed. This phenomenon has only been observed for non-autonomous oscillators, and only when examined over finite time periods. When observed in an asymptotic, averaging time scale, it can easily be mistaken for complete desynchronization.

For living systems, synchronization between oscillators represents a state of stability and cooperative working between oscillators. Synchronized oscillators are, to an extent, able to resist perturbation away from this state and coordinate their oscillations for a variety of ends, including temporally compartmentalizing conflicting processes (Tu et al., 2005; Lloyd et al., 2018). As in, for example, Lancaster et al. (2016), certain combinations of synchronization can be considered as the “healthy” state of a cell, and the parameters at which they do and do not exist can therefore inform us about the mechanisms of pathological transitions. We will apply these methods of synchronization analysis to our cellular metabolism model.

### 2.4. Numerical Simulations

We conducted analysis of the model to determine the impacts on the dynamics made by the additions of weighted networks and deterministic non-autonomicity to the Lancaster et al. (2016) model. These simulations involved numerical integration of the differential phase equations, defined in Equation (7). This was conducted using the inbuilt Matlab ode15s algorithm, which is a partially implicit numerical integration scheme using a variable integration step and evaluates errors through interpolated backwards differences (Shampine and Reichelt, 1997). The equations were integrated for a period of 10,000 s at a sampling frequency of 0.1 s. The first 5,000 s were discarded, assuming they were dominated by transient dynamics, and then the final 5,000 s analyzed to determine what, if any, modes of synchronization were present.

This analysis involved calculating the phase coherence, as defined in Bandrivskyy et al. (2004) and Sheppard et al. (2012), between the glycolysis and OXPHOS oscillators and their glucose and oxygen drivers, respectively, and between the network oscillators and the mean field driving of the other network. The phase difference between these components was also calculated, as was the Kuramoto order parameter of each network.

For autonomous systems, time series are defined as coherent at a phase coherence value of or close to 1. However in non-autonomous systems, series may be coherent yet exhibit a time-averaged phase coherence of significantly less than 1 due to their modulation in time away from their coherent mean. Additionally, slight numerical simulation errors and noise can make it impossible to attain a numerical phase coherence of precisely 1. Through observations of numerical simulations, we have therefore defined coherence greater than 0.9 and phase difference within a bounded 2π region for the entire 5,000 s as indicative of permanently synchronized oscillators. If the coherence value was greater than 0.9 but the phase difference unbounded, we instead categorized the oscillators as intermittently synchronized. Networks were considered synchronized when their time-averaged Kuramoto order parameter exceeded 0.5, the threshold at which a network is more ordered than disordered. This was considered permanent if the parameter varied by less than 0.2 over the entire 5,000 s, and intermittently if it varied by more than this. Similarly to phase coherence, the Kuramoto order parameter of non-autonomous oscillations will naturally vary in time due to frequency modulation, even in highly ordered networks, and so simulations indicated that only variations of greater than 0.2 are due solely to intermittency or disorder.

### 2.5. Experimental Comparison

We have also analyzed data collected by Amemiya et al. (2017) on glycolytic oscillations of starved HeLa cells. In this experiment, the optical NADH fluorescence of numerous cells was measured over time after glucose was added to their environment. We calculated the group phase coherence, as defined by Sheppard et al. (2016), of groups of cells around the culture. This coherence was further tested against 19 WIAAFT surrogates, as defined in Lancaster et al. (2018), such that any non-zero coherence is considered significant. We analyzed both groups near to one another and far from one another, to identify any significant differences between the two. These groups were constructed using hierarchical agglomerative clustering with the “complete” linkage method, which considers the furthest Euclidean distance between groups of cells when defining the clusters. The culture was 1400μ*m* by 1200μ*m* in area, and near groups were defined as having 300–400μ*m* between their average positions, while the average positions of far groups were 900–1, 200μ*m* apart.

Simulations of this experiment were also conducted, using some of the results of the group coherence analysis and the general numerical simulations. This was done by numerically integrating a realization of the system at a certain parameter set using a four step Runge-Kutta algorithm. The results of this and all the above methods are presented in the following section.

## 3. Results

There are six possible modes of synchronization within our cellular metabolism model: glycolysis to glucose, glycolysis network, glycolysis to OXPHOS, OXPHOS network, and OXPHOS to oxygen. While it would not be possible for glycolysis to be synchronized to OXPHOS, but OXPHOS to not synchronize to glycolysis in an individual oscillator model, it is possible for a network to become synchronized to a mean field driving, without the network from which that mean field arises becoming synchronized to the network it is driving.

We examined whether each of these synchronizations occurred, and whether they were permanent or intermittent, at 2,500 different combinations of the parameters *F*_*GO*_ and *F*_*MO*_, as defined in Equation (2). This is similar to the analysis conducted in Lancaster et al. (2016), and hence provides some understanding of the impact of each of the changes we have made in this model.

The parameters for which these simulations were conducted are given in Table 2. Most of these parameters, ϵ_*G*_, ϵ_*O*_, *F*_*GO*_, *F*_*MO*_, ω_*G*_, ω_*GO*_, ω_*MO*_, ω_*O*_, are identical to those used in Lancaster et al. (2016) to allow a direct comparison, revealing the effects of the changes from that model. *K*_*GO*_ and *K*_*MO*_ did not exist in the Lancaster et al. model, and they have been set to be equal to the other non-varied coupling parameters. The frequencies and amplitudes of modulation were determined by their ratio to the mean frequencies, as studied by Lucas et al. (2019). *W* may be set to 1 as the relevance of the weighted coupling is in the relative weighings between different oscillator pairs. *N* and *M* cannot be determined purely biologically: the glycolysis oscillators represent a collection of often-distributed glycolytic enzymes that are not realistically quantifiable, while the number of a mitochondria in a cell type can vary significantly (Wilson, 2017; Chaudhry and Varacallo, 2020). Instead, the network sizes are chosen such that there a sufficiently many oscillators to validate the mean field approximation (Strogatz and Mirollo, 1991), and not so many as to make computational simulation infeasible.

**Table 2:** Parameters of the non-autonomous weighted network simulations.

**Parameter**	**Value(s)**
ϵ_*G*_	0.025
ϵ_*O*_	0.025
*K*_*GO*_	0.025
*K*_*MO*_	0.025
*F*_*GO*_	[0, 0.3]
*F*_*MO*_	[0, 0.3]
ω_*G*_	$\frac{2\pi }{200}$ Hz
ω_*GO*_	$\frac{2\pi }{200}$ Hz
ω_*MO*_	$\frac{2\pi }{100}$ Hz
ω_*O*_	$\frac{2\pi }{100}$ Hz
ω_*Gm*_	$\frac{2\pi }{2000}$ Hz
ω_*GOm*_	$\frac{2\pi }{2000}$ Hz
ω_*MOm*_	$\frac{2\pi }{1000}$ Hz
ω_*Om*_	$\frac{2\pi }{1000}$ Hz
*A*_*G*_	$\frac{2\pi }{600}$ Hz
*A*_*GO*_	$\frac{2\pi }{600}$ Hz
*A*_*MO*_	$\frac{2\pi }{300}$ Hz
*A*_*O*_	$\frac{2\pi }{300}$ Hz
*N*	100
*M*	100
*W*	1

**Figure 4:**
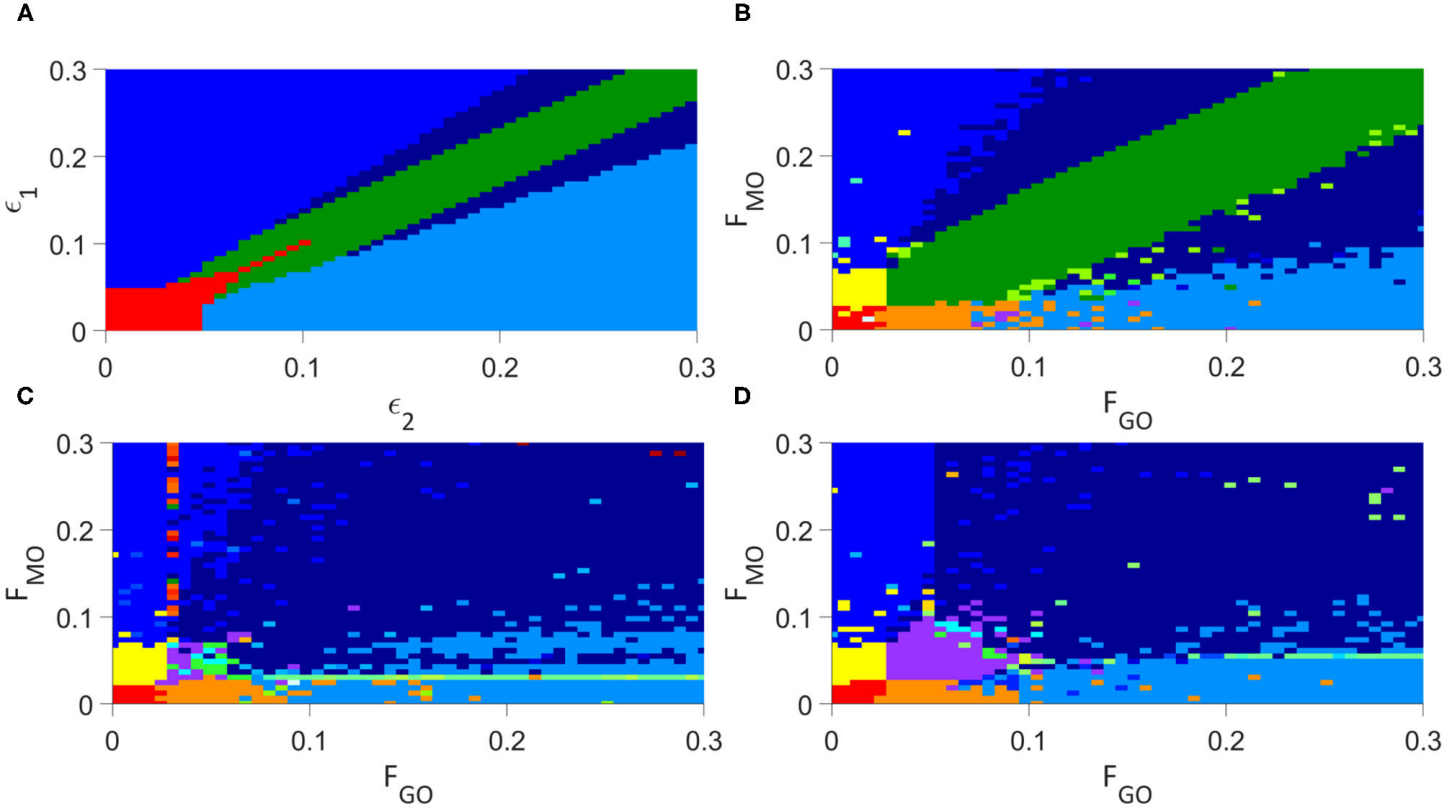
Analysis of the synchronization regimes at different parameter values, at parameter steps of 0.006 between each simulation, **(A)** for the modified Lancaster et al. (2016) model. **(B)** for the Lancaster et al. individual oscillator model with added deterministic non-autonomous frequencies and intermittent synchronization analysis. **(C)** for the unweighted network model. **(D)** for the weighted network model. Regimes are defined in Table 3.

We present first the analysis of the individual oscillator model of Lancaster et al. (2016) in Figure 4A, for parameters ϵ_2_ and ϵ_1_ as defined in Equation (1).

Introducing each new element of our model in turn to examine this same parameter space, we first include our deterministic variation of the frequency and analyse for intermittent synchronization, as well as permanent, but otherwise maintain the Lancaster et al. (2016) model. The results are in Figure 4B, and the main regimes described in Table 3. This results in the splitting of the red region in the Lancaster et al. (2016) analysis into three regimes, two new: permanent synchronization between glycolysis and glucose only, and oxygen and OXPHOS only. The dark blue region, where only glycolysis and OXPHOS are synchronized, is also made significantly larger, and there are spots of intermittently synchronized regimes that appear only briefly throughout the parameter space.

**Table 3:** Synchronization regimes for each simulated model.

**Model**	**Color**	**GO-G**	**GO**	**GO-MO**	**MO-GO**	**MO**	**MO-O**
Non-network models	Red	Permanent	N/A	No	No	N/A	Permanent
	Yellow	Permanent	N/A	No	No	N/A	No
	Blue	Permanent	N/A	Permanent	Permanent	N/A	No
	Orange	No	N/A	No	No	N/A	Permanent
	Dark green	No	N/A	No	No	N/A	No
	Dark blue	No	N/A	Permanent	Permanent	N/A	No
	Light blue	No	N/A	Permanent	Permanent	N/A	Permanent
Network models	Red	Permanent	Permanent	No	No	Permanent	Permanent
	Orange	No	Permanent	No	No	Permanent	Permanent
	Light blue	No	Permanent	Permanent	Permanent	Permanent	Permanent
	Yellow	Permanent	Permanent	No	No	Permanent	No
	Blue	Permanent	Permanent	Permanent	Permanent	Permanent	No
	Purple	No	Permanent	No	No	Permanent	No
	Green	No	Permanent	No	No	Intermittent	No
	Cyan	No	Intermittent	No	No	Permanent	No
	Dark blue	No	Permanent	Permanent	Permanent	Permanent	No

The next step is to introduce unweighted networks of glycolysis and OXPHOS oscillators. The result is in Figure 4C. This introduces a new regime, where only the networks are internally synchronized, and converts the dark green regime, where there is no synchronization, into the even further increased dark blue regime. Once again, intermittent regimes are spotted briefly throughout the parameter space.

The final step in constructing our full model, is to weight the glycolysis and OXPHOS networks according to Equations (3) and (4). Figure 4D shows the results of this final simulation. This splits the new regime observed in the previous simulation into the purple, green and cyan regimes: the purple representing the same permanent synchronization within each network, the green a new intermittent synchronization of the OXPHOS network, and the cyan a new intermittent synchronization of the glycolysis network. The weighting reduces the size of the dark blue region, giving more space to the blue and light blue, and as in the previous simulations produces small regimes of intermittent synchronization.

### 3.1. Experimental Comparison

In Amemiya et al. (2019) constructed a model of cellular glycolysis to explain the glycolytic oscillations they had observed in HeLa cells. This model adopted an approach more similar to the mainstream discussed in the previous section. We therefore offer a comparison between this model and the one we have presented here, to help illuminate further the differences between our approach and ones more characteristic of the cellular modeling mainstream, applied in the context of this experiment.

The Amemiya et al. (2019) model constructs glycolysis as two main processes: the phosphofructokinase 1 (PFK) reaction and the pyruvate kinase (PK) reaction. The former is modeled as the first step, converting glucose and ATP into intermediaries, while the second is the last reaction, converting these intermediaries into ATP and pools of NADH and other products. The model focuses on the masses of the metabolites required for these reactions, from their entry into the cell to their consumption in the metabolic process. This technique consists of seven autonomous linear differential equations and twenty two parameters to model the glycolysis metabolic branch only, which contrasts to the four non-autonomous non-linear oscillator equations of Equation (7) and the thirteen parameters of Table 2 to model both the glycolysis and OXPHOS branches.

In addition to a measure of coherence within a network, the order parameter may also be considered the amplitude of the network's mean field. We can therefore consider it both an indication of the amplitude of our system, and the degree to which the glycolysis and OXPHOS networks are operating effectively. We introduce a modified Kuramoto order parameter *s*, where

$$se^{i{\text{Ψ}}_{GOMO}}=\frac{1}{\left(N+M\right)}\left(\overset{N}{\underset{i=1}{\sum }}e^{i\theta_{GOi}}+\overset{M}{\underset{j=1}{\sum }}e^{i\theta_{MOj}}\right),$$

which takes into account both networks. This parameter can be compared to the time series of NADH fluorescence from a single cell in the Amemiya et al. (2017) experiment, as NADH production in the cellular metabolic system is maximized when glycolysis and OXPHOS are able to act coherently. We provide this comparison in **Figure 6**, and this can be further compared to an equivalent output of the model in Figure 2 of Amemiya et al. (2019). The Amemiya et al. (2019) model considers just glycolysis, and is built on 7 autonomous differential equations tracking the change in quantities of a range of metabolites, relying on 22 parameters In contrast, the model we have presented here accounts for both glycolysis and OXPHOS through two types of non-autonomous differential phase equations, using 21 parameters.

**Figure 5:**
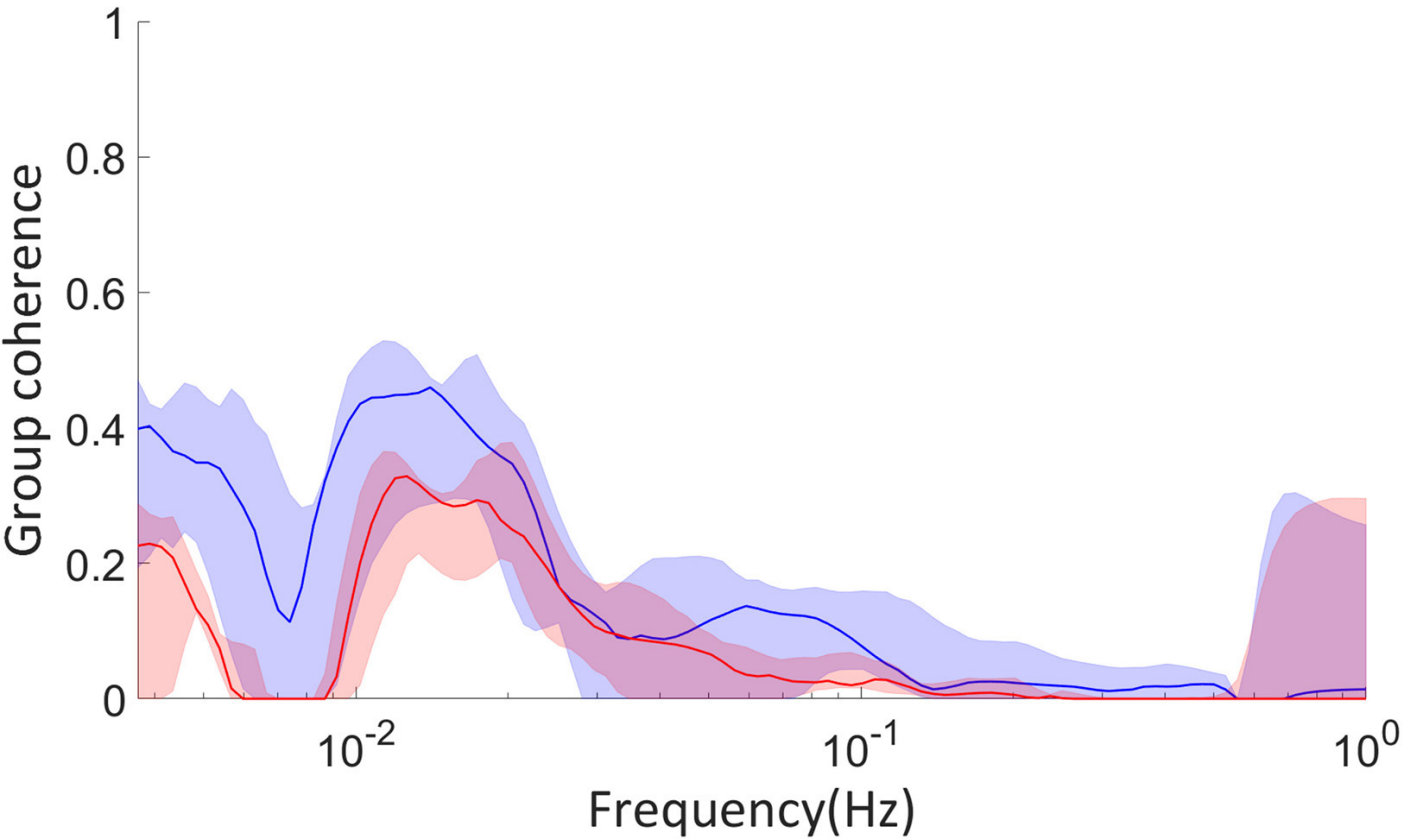
Surrogate tested coherence between groups of cells examined in Amemiya et al. (2017), calculated with the coherence algorithm presented in Sheppard et al. (2016). Red coloring indicates groups of cells far from one another, 900–1, 200μ*m* distance between their average positions, and blue close to one another, 300–400μ*m* distance between their average positions. The dimensions of the culture were 1, 400 by 1, 200μ*m*. The solid colored lines are the median coherence of each pair of groups, and the shaded regions the range from the minimum to maximum coherence. The cell groups were constructed using hierarchical agglomerative clustering with the “complete” linkage method.

**Figure 6:**
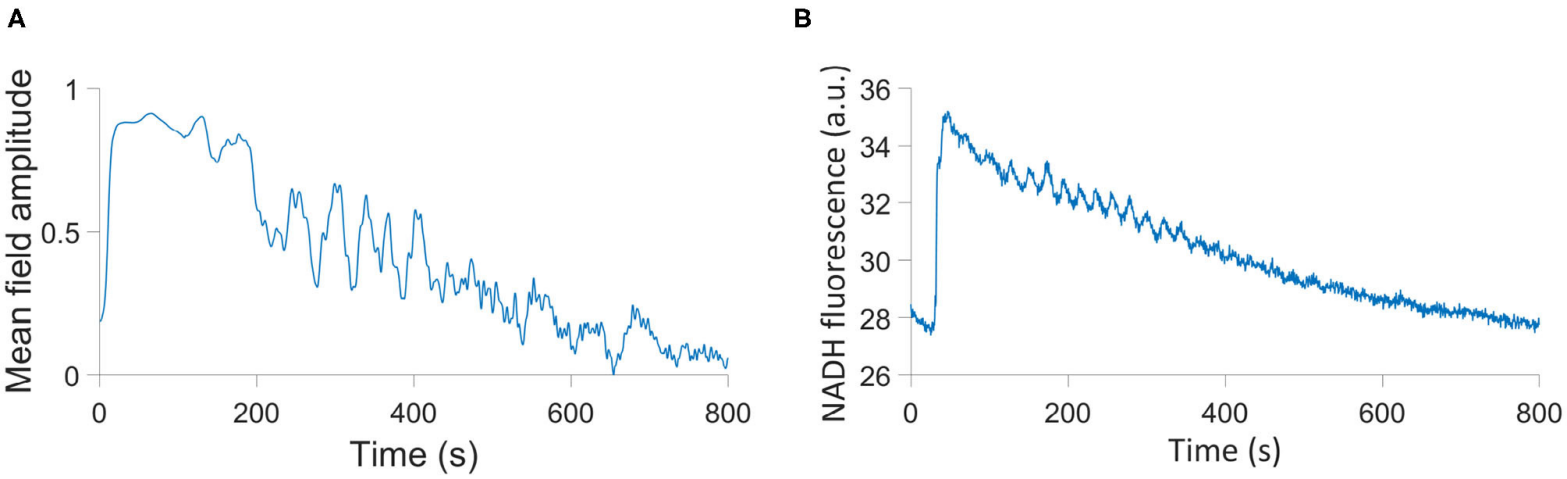
**(A)** Simulation of the HeLa experiment using a modified order parameter. **(B)** The time series of NADH fluorescence in a single cell in the Amemiya et al. (2017) experiment.

The parameters used in this simulation are given in Table 4, where *A* = 9.511 × 10^−7^ and *B* = 1.931 × 10^−3^ are the coefficients of the quadratic and linear terms, respectively, of the curve in **Figure 6B**, as found by quadratic curve fitting. The modulation frequency of the glycolysis oscillations was extracted from group coherence analysis of the Amemiya et al. data, which found that for both cell groups close to and far from one another there was significantly coherent oscillations in the range 0.01–0.02 Hz. This analysis is presented in Figure 5. The other frequencies were selected to maintain the same ratio with the extracted glycolysis modulation as discussed in Lancaster et al. (2016). The coupling parameters were chosen to reflect the dynamics shown in the experimental time series and identified in Figure 4: the simulation begins with *F*_*GO*_ = *F*_*MO*_ = 0.6, and all other parameters at 0.025 to re-create the dark blue regime of synchrony between the networks found in Figure 4D, resulting in the initial amplitude spike as glucose is first introduced to the environment. Over the next 355.9s these couplings decrease according to the gradient of Figure 6B and ϵ_*GO*_ equivalently increases, as the damage the cells sustained during their starvation period inhibits their processes and reducing metabolite supplies leaves the system less stable to fluctuations in these quantities. This results in a trending decrease in the networks' amplitude and the emergence of oscillations. After 382.9 s the supply of glucose is almost entirely exhausted, flat-lining ϵ_*GO*_ at 0.7 and causing the oscillations to begin to degrade into more noise-like behavior. For the final 517s of the simulation *F*_*GO*_ and *F*_*MO*_ have reached 0 as the cells begin to die, their oscillations continue to diminish, and their NADH production dries up.

**Table 4:** Parameters of the HeLa experiment simulation.

**Parameter**	**Value(s)**		
	**0–355.9 s**	**356–382.9 s**	**383–800 s**
ϵ_*G*_	−*At*^2^+*Bt*+0.025	0.7	0.7
ϵ_*O*_	0.025	0.025	0.025
*K*_*GO*_	0.025	0.025	0.025
*K*_*MO*_	0.025	0.025	0.025
*F*_*GO*_	*At*^2^−*Bt*+0.6	*At*^2^−*Bt*+0.6	0
*F*_*MO*_	*At*^2^−*Bt*+0.6	*At*^2^−*Bt*+0.6	0
ω_*G*_	$\frac{3\pi }{10}$Hz	$\frac{3\pi }{10}$Hz	$\frac{3\pi }{10}$Hz
ω_*GO*_	$\frac{3\pi }{10}$Hz	$\frac{3\pi }{10}$Hz	$\frac{3\pi }{10}$Hz
ω_*MO*_	$\frac{3\pi }{5}$Hz	$\frac{3\pi }{5}$Hz	$\frac{3\pi }{5}$Hz
ω_*O*_	$\frac{3\pi }{5}$Hz	$\frac{3\pi }{5}$Hz	$\frac{3\pi }{5}$Hz
ω_*Gm*_	$\frac{3\pi }{100}$Hz	$\frac{3\pi }{100}$Hz	$\frac{3\pi }{100}$Hz
ω_*GOm*_	$\frac{3\pi }{100}$Hz	$\frac{3\pi }{100}$Hz	$\frac{3\pi }{100}$Hz
ω_*MOm*_	$\frac{3\pi }{50}$Hz	$\frac{3\pi }{50}$Hz	$\frac{3\pi }{50}$Hz
ω_*Om*_	$\frac{3\pi }{50}$Hz	$\frac{3\pi }{50}$Hz	$\frac{3\pi }{50}$Hz
*A*_*G*_	$\frac{3\pi }{30}$Hz	$\frac{3\pi }{30}$Hz	$\frac{3\pi }{30}$Hz
*A*_*GO*_	$\frac{3\pi }{30}$Hz	$\frac{3\pi }{30}$Hz	$\frac{3\pi }{30}$Hz
*A*_*MO*_	$\frac{3\pi }{15}$Hz	$\frac{3\pi }{15}$Hz	$\frac{3\pi }{15}$Hz
*A*_*O*_	$\frac{3\pi }{15}$Hz	$\frac{3\pi }{15}$Hz	$\frac{3\pi }{15}$Hz
*N*	100	100	100
*M*	100	100	100
*W*	1	1	1

While the curve presented in Figure 6A depends on the initial phases of each oscillator, which are randomized, and therefore will not be identical from simulation to simulation, its oscillator features and overall trend are indicative of the parameters in Table 4. And while this simulation is not an identical reflection of the experiment in every feature, it is an indication of the capacity of our model to reproduce the oscillating nature of biological processes, and the ease with which it can be adapted to a plethora of different cells and circumstances.

## 4. Discussion

The conversion of established metabolic models, such as that of Lancaster et al. (2016), to consider networks of processes offers both greater biological realism and a resulting transformation of the dynamics we expect to see from such models. The step from Figures 4B,C for example overhauls the parameter space, introducing entirely new regimes and destroying once-firm fixtures of the non-network model. It is clear from all of these results that networks result in an even greater area of the parameter space featuring synchronization, with the only regime of total desynchronization disappearing once networks are introduced, and the networks themselves never being desynchronized. This aligns well with the imperative of such biological processes to remain robust against significant external perturbations, and the expectation that these parameter values do not represent catastrophic departure from the healthy state of the system. More significant perturbations of the coupling parameters, to both higher values and the entire elimination of more coupling modes, are likely required to completely desynchronize the networks, which would represent even further departures from the healthy parameter states of the cell.

In healthy human cells, ATP is produced primarily through OXPHOS, with support from glycolysis. In our model, this may be represented by synchronization between the networks, and between the OXPHOS network and its oxygen driving (Lancaster et al., 2016). Internal synchronization of both networks is also required to characterize a healthy condition: disregulation within the metabolic processes is a key indicator of a malfunctioning cell. This state is represented in the bottom right of each graph in Figure 4, but is significantly diminished in area with the addition of deterministic frequency modulation from Figure Figures 4A,B. A cancerous state, may be indicated by an opposite state: a mode switch to the dominance of glycolysis, known as the Warburg effect, is reflected by synchronization between the networks and between glycolysis and glucose, but not OXPHOS and oxygen (Lancaster et al., 2016). Due to the decreased relevance of OXPHOS to the metabolic process in cancer, it may be represented by either ordered or disordered OXPHOS networks. This regime is found in the top left of each of Figure 4, similarly decreasing in area between Figures 4A,B as with the bottom right regime.

Network models also offer greater potential for oscillator systems: while reducing oscillating differential equations to just their phase provides a much simpler system that still contains the key dynamics, only at the mesoscopic level of networks of many oscillators can the system amplitude be rebuilt. Further work on this model could therefore provide not just an order parameter of the network indicative of its activity, but an amplitude of its production.

The turn to deterministic non-autonomous frequencies and finite time synchronization analysis similarly promises a significant change to the dynamics of metabolic models. Intermittent synchronization allows greater nuance between the states of “healthy” and “pathological,” more reflective of the complexity of living systems, yet further ways for the processes to stabilize in spite of significant perturbation and ever more complex and effective ways for them to compartmentalize. However, with the introduction of this non-autonomicity comes greater challenges for numerical simulations: the numerical integration of non-linear oscillating differential equations is an already delicate task, and the addition of another dimension of time sensitivity requires alternative methods.

Further work with more sensitive numerical integration algorithms and more sophisticated methods for identifying intermittent synchronization would be likely to find a far greater role of the phenomenon in the model's parameter spaces, and further clarify exactly which dynamic we can expect to find at each parameter combination. The integration scheme used in this work has resulted in multiple “islands” of synchronization regimes, which are unrelated to the regimes at all neighboring parameter values, and yet are reproduced under the same simulation conditions. Non-autonomous oscillations pose a particular challenge to numerical integration schemes due to their two highly distinct frequency modes. Schemes designed to adapt to this situation may be able to provide greater clarity on our model, with which we may be able to further identify parameters leading to pathological states and more complex dynamics within the model.

## Data Availability Statement

The measured data analyzed in this paper were originally collected and presented by Amemiya et al. (2017). They are available at doi: 10.17635/lancaster/researchdata/406. The MatLab codes used for numerical modeling and analyses of the numerical and measured data can be found at doi: 10.17635/lancaster/researchdata/409.

## Author Contributions

JRA proposed the inclusion of weighted networks, designed and ran the numerical simulations of the model, performed thorough investigation of the model behavior including the intermittent synchronization analysis, and drafted the manuscript. AS conceived the study, and proposed to incorporate networks instead of individual oscillators used in the earlier version of the model. She oversaw the development of the work, provided regular guidance at every stage, and advised about the structure and the content of the manuscript. All authors edited the manuscript and approved the submitted version.

## Conflict of Interest

The authors declare that the research was conducted in the absence of any commercial or financial relationships that could be construed as a potential conflict of interest.
